# Association between *RIT2* rs16976358 Polymorphism and Autism Spectrum Disorder in Asian Populations: A Meta-analysis

**DOI:** 10.1155/2023/8886927

**Published:** 2023-02-11

**Authors:** Jing Wang, Shoupeng Wei, Jin Zhang, Hu Wang

**Affiliations:** ^1^Department of Cellular and Molecular Medicine, University of California San Diego, 9500 Gilman Drive, La Jolla, CA 92093, USA; ^2^Laboratory for Integrative Neuroscience, National Institute on Alcohol Abuse and Alcoholism, National Institutes of Health, Bethesda, MD 20807, USA; ^3^State Key Laboratory of Chemical Oncogenomics, Guangdong Provincial Key Laboratory of Chemical Genomics, Shenzhen Graduate School, Peking University, Shenzhen 518055, China; ^4^Institute of Cell Engineering, School of Medicine, Johns Hopkins University, Baltimore 21215, USA

## Abstract

**Background:**

Recent studies have shown that Ras-like without CAAX2 (*RIT2*) polymorphism is a susceptible factor for Parkinson's disease (PD) and autism spectrum disorder (ASD). SNP rs12456492 and rs16976358 show the emerging evidence of increased risk of PD and ASD, respectively. A meta-analysis examining the relationship between rs12456492 and PD was reported, but the association between rs16976358 and ASD has not been investigated.

**Methods:**

We searched literature from the databases PubMed, Embase, Google Scholar, ScienceDirect, EBSCOhost, OVID, Web of Science, and Wiley up to February 2021. Three studies including 1160 ASD cases and 1367 controls were eventually enrolled in the meta-analysis based on strict inclusion and exclusion criteria.

**Results:**

All genetics models indicate a significant association between rs16976358 polymorphism and ASD susceptibility (C vs. T: *p* = 0.001; CC vs. TT: *p* = 0.001; CT vs. TT: *p* = 0.009; CC+CT vs. TT: *p* = 0.001; CC vs. CT+TT: *p* = 0.001; TT+CC vs. CT: *p* = 0.013). The results of sensitivity analysis and publication bias of Begg's and Egger's tests were stable in the models of allele (C vs. T), codominant (CC vs. TT), dominant (CC+CT vs. TT), and recessive (CC vs. CT+TT).

**Conclusions:**

Our meta-analysis exhibits that the allele C, CC, and CT genotyping of rs16976358 suggest the risk for ASD, but additional studies using a large sample size and ethnically diverse populations need to be included in the future.

## 1. Introduction

Autism spectrum disorder (ASD) is a neurodevelopmental disorder characterized by global developmental delay, speech and language impairment, intellectual disability, restricted interests, and social communication deficits. It affects approximately 1-2% of the population worldwide with a ratio of 3 : 1 in males compared with females [1]. The etiology of ASD is diverse and complex, affected by both genetic and environmental factors. Since the 2000s, genetic studies have revealed that the genetic traits leading to ASD were multigenic and strongly heterogeneous. Many susceptible genomic regions were identified by a large-scale genome sequencing following single nucleotide polymorphism (SNP) studies. More SNPs in variant genes were revealed to be associated with ASD, such as microtubule affinity-regulating kinase 1 (*MARK1*), oxytocin receptor (*OXTR*), SH3 and multiple ankyrin repeat domains 3 (*SHANK3*), and gamma-aminobutyric acid type A receptor subunit beta3 (*GABRB3*) [2–5]. However, these mutations only partially account for ASD pathomechanism. It is still of importance to investigate other genes, which may contribute to the risk of ASD.

Recently, Ras-like without CAAX2 (*RIT2*) has been introduced as a genetic risk gene for neurological and psychiatric disorders such as Parkinson's disease (PD), schizophrenia, and ASD. RIT2, as a member of the Ras superfamily, is highly expressed in the brain, in particular the retinal ganglion cells and dopaminergic neurons [6, 7]. It acts as GTPases and regulates the intracellular signaling cascades in neuronal differentiation, neurogenesis, neurite growth, and branching [8]. In 2012, a novel locus, *RIT2* rs12456492, was identified as a risk factor for PD patients in the white population by conducting a meta-analysis of genome-wide association study (GWAS) [9]. This rs12456492 SNP is also confirmed based on Asian populations with Parkinson's disease [10]. In 2016, Liu et al. found that, in a Japanese and Han Chinese populations, a *RIT2* rs16976358 polymorphism had the strongest association with ASD [11], suggesting that RIT2 polymorphism also indicates the risk of ASD. To drive a precise estimation of the association between rs16976358 and ASD, we perform a meta-analysis to investigate the role of rs16976358 in the pathogenesis of ASD, which provides broad possibilities for further studies.

## 2. Methods

### 2.1. Literature Search

The databases PubMed, Embase, Google Scholar, ScienceDirect, EBSCOhost, OVID, Web of Science, and Wiley were used for the literature search. The searching strategy was applied by the following terms: “Autism spectrum disorder or autism or ASD” and “Ras-like without CAAX 2 or RIT2 or Rin or rs16976358” and “polymorphism or SNP”, with the last report up to February 26th, 2021. In addition, we also examined the references in the retrieved papers to identify extra studies.

### 2.2. Inclusion and Exclusion Criteria

The inclusion criteria are listed as follows: (1) association between rs16976358 and ASD risk; (2) case-control studies; (3) those providing sufficient genotyping data to calculate the odds ratio (OR) and 95% confidence interval (CI); and (4) patients with ASD were diagnosed according to the Diagnostic and Statistical Manual of Mental Disorders (5th edition) or the Autism Diagnostic Inventory-Revised (ADI-R) criteria. Studies were excluded for the following reasons: (1) republished or duplicated studies; (2) animal tests or reviews or nonrelated; and (3) insufficient genotyping data.

### 2.3. Data Extraction

Data were collected independently by two investigators according to the inclusion and exclusion criteria described above. In each study, the following information was extracted from the eligible studies: first author, year of publication, study type, region, ethnicity of the sample population, genotyping method, sample size of case, and control groups. Genotyping distributions and minor allele frequency (MAF) were separately listed to facilitate analysis. For the data which are not provided in published papers or the supplementary materials, the relevant information was obtained by direct communications with the corresponding authors.

### 2.4. Statistical Analysis

Statistical analyses were performed by STATA software, version 15.0 (STATA Corp., College Station, TX, USA). The association between rs16976358 and risk of ASD was evaluated by pooled OR and 95% CI. Six genetic models were established for analysis, including allele (C vs. T), codominant (CC vs. TT), codominant (CT vs. TT), dominant (CC+CT vs. TT), recessive (CC vs. CT+TT), and over dominant (TT+CC vs. CT). The significance of OR was determined by the *Z*-test, in which *p* < 0.05 was considered as statistically significant. We examined the heterogeneity with the *Q*-test and *I*^2^ statistics using the fixed- and random-effect model. If *P*_*Q*_ > 0.10, the fixed-effect model was adopted to calculate the pooled ORs; if *P*_*Q*_ < 0.10, the random-effect model was applied instead. Sensitivity analysis was carried out by sequentially omitting one study at a time to estimate the stability of the results. Publication bias among studies was determined using funnel plot and Begg's and Egger's tests. A meta-regression analysis was used to assess the impact on moderator covariables on the heterogeneity of results.

## 3. Results

### 3.1. Study Characteristics

**Figure 1:**
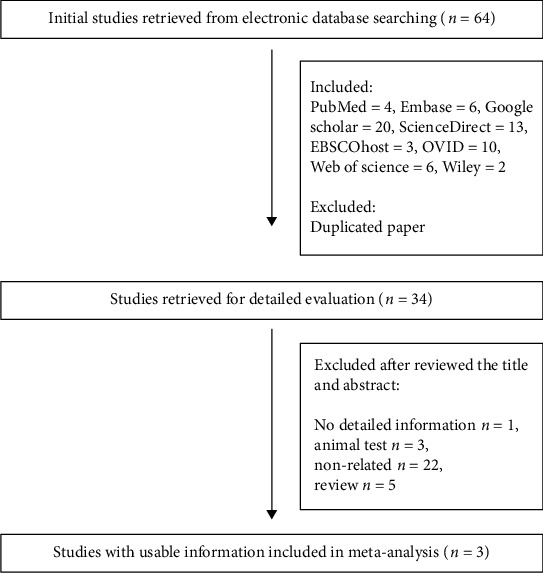
Flow diagram of study selection.

Sixty-four initial studies were retrieved from the databases (PubMed: 4, Embase: 6, Google Scholar: 20, ScienceDirect: 13, EBSCOhost: 3, OVID: 10, Web of Science: 6, and Wiley: 2). Thirty-four studies were remained by removing duplicates. After browsing through the titles and abstracts, we excluded 5 reviews, 3 animal tests, 22 irrelevant studies, and 1 research article because of sufficient genotyping information. Finally, three studies published between 2016 and 2017 were selected with a collection of 1160 ASD cases and 1367 controls (Figure 1). All of these three studies were carried out in Asian populations. The characteristics and genotyping distribution are separately listed in Tables 1 and 2.

### 3.2. Meta-analysis Results

**Figure 2:**
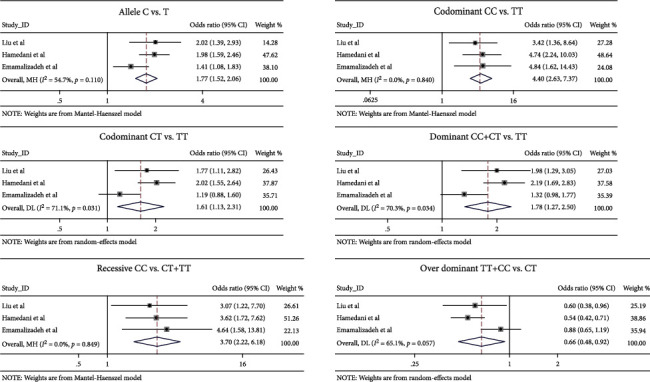
Forest plots for meta-analysis of rs16976358 polymorphism and risk of ASD in Asian populations in six genetic models ((the allele (C vs. T), codominant (CC vs. TT), codominant (CT vs. TT), dominant (CC+CT vs. TT), recessive (CC vs. CT+TT), and over dominant (TT+CC vs. CT)). Allele (C vs. T), codominant (CC vs. TT), and recessive (CC vs. CT+TT) were analyzed by fixed model. Codominant (CT vs. TT), dominant (CC+CT vs. TT), and over dominant (TT+CC vs. CT) were assessed by random model. *Z*-test was used for evaluating *p* value; *p* < 0.05 was considered as statistically significant.

The association between rs16976358 and ASD risk was assessed by 6 genetic models, including allele (C vs. T), codominant (CC vs. TT), codominant (CT vs. TT), dominant (CC+CT vs. TT), recessive (CC vs. CT+TT), and over dominant (TT+CC vs. CT). Odds ratio (OR), 95% confidence interval (CI), and *p* values from these models in the studies were, respectively, shown in Table 3. Meta-analysis was performed on the combined population by the fixed-effect or random-effect model based on the heterogeneity. The heterogeneity was significant in models of codominant (CT vs. TT) and dominant (CC+TT vs. TT) (*P*_*Q*_ < 0.05). *I*^2^ statistics showed that all the genetic models had high heterogeneity (*I*^2^ > 50%) (Table 4). In overall population, we found that rs16976358 was significantly associated with ASD risk in all the genetic models (C vs. T: OR = 1.767, 95% CI: 1.517-2.057, *p* = 0.001; CC vs. TT: OR = 4.402, 95% CI: 2.630-7.369, *p* = 0.001; CT vs. TT: OR = 1.614, 95% CI: 1.127-2.311, *p* = 0.009; CC+CT vs. TT: OR = 1.781, 95% CI: 1.270-2.498, *p* = 0.001; CC vs. CT+TT: OR = 3.700, 95% CI: 2.217-6.176, *p* = 0.001; TT+CC vs. CT: OR = 0.663, 95% CI: 0.479-0.916, *p* = 0.013) (Figure 2). Thus, the C allele and CC and CT genotyping of rs16976358 polymorphism indicate ASD susceptibility.

### 3.3. Metaregression and Sensitive Analysis

**Figure 3:**
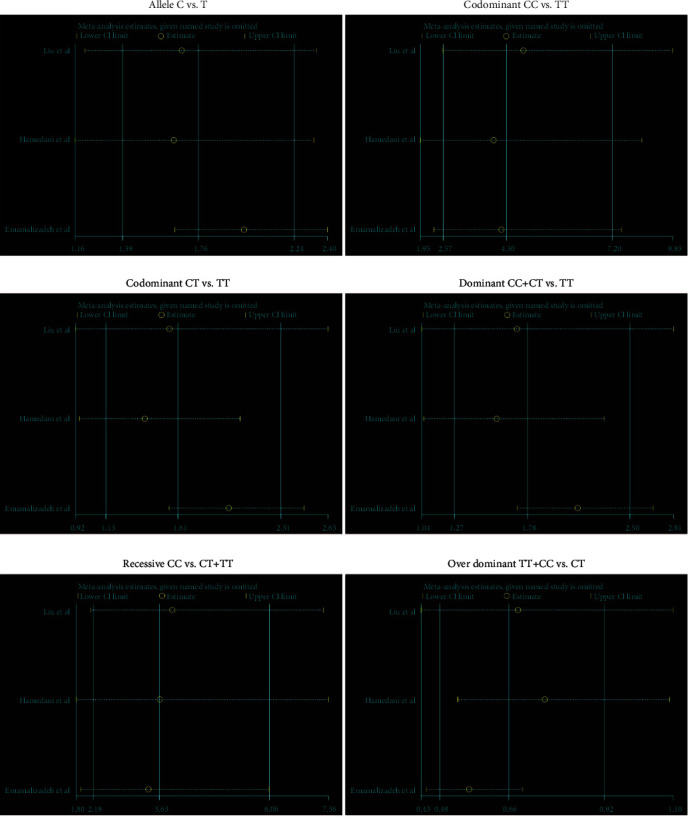
Sensitive analysis of rs16976358 polymorphism and risk of ASD in the six genetic models. Meta-analysis was estimated by omitting one given named study. OR lower and upper CI limit were listed to show the range of estimated values.

Metaregression was performed to explore the effect of covariables on the heterogeneity caused in our study. Different publication years and regions had no impacts on the heterogeneity found in any genetic models (Table 5). Unfortunately, we do not have sufficient data to analyze other factors contributing to the heterogeneity. To estimate the stability of the results, sensitivity analysis was carried out in the 6 genetic models. In Figure 3, pooled OR and 95% CI values were not influenced by sequentially omitting one study at a time, may suggesting the high stability of this meta-analysis. To analyze it in detail, we found that the results in the codominant (CT vs. TT) were unstable after omitting Liu et al. (estimated OR = 1.557, 95% CI = 0.922-2.629, *p* = 0.097) and Hamedani et al.' s studies (estimated OR = 1.389, 95% CI = 0.948-2.036, *p* = 0.092). In the model of over dominant (TT+CC vs. CT), sensitivity was also unstable after deleting Liu et al. (estimated OR = 0.687, 95% CI = 0.430-1.099, *p* = 0.118) and Hamedani et al.'s studies (estimated OR = 0.758, 95% CI = 0.527-1.090, *p* = 0.135). But there was high sensitivity in the remained models of allele (C vs. T), codominant (CC vs. TT), dominant (CC+CT vs. TT), and recessive (CC vs. CT+TT) (*p* < 0.05) (Table 6).

### 3.4. Publication Bias

**Figure 4:**
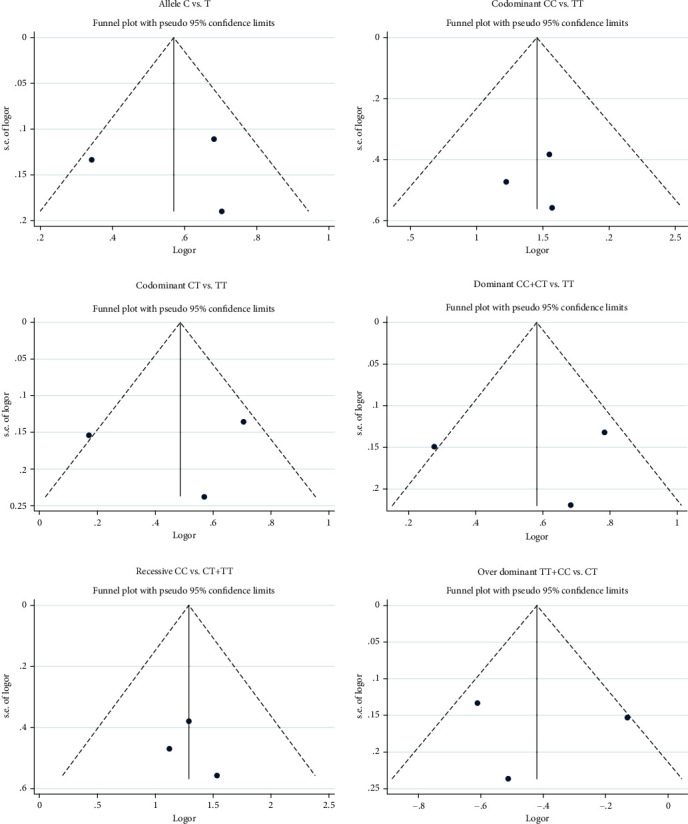
Funnel plot was performed in the genetic models to show publication bias in this meta-analysis.

Funnel plot and Begg's and Egger's tests were carried out to evaluate the publication bias in our study. A publication bias was found in codominant (CT vs. TT) and dominant (CC+CT vs. TT) models in funnel plot. One paper (Emamalizadeh et al.) was slightly apart from the symmetrical shape in these 2 models (Figure 4). However, no publication bias was shown in Begg's and Egger's tests (Table 7). Based on these results, the number of studies analyzed in our current meta-analysis may be not sufficient.

## 4. Discussion

In two distinct GWAS reports, *RIT2* gene was identified as a new locus for both Parkinson's disease (PD) and autism spectrum disorder (ASD) [9, 11, 12]. SNP analysis results in each study showed that rs12456492 and rs16976358 were associated with the risk of PD and ASD, respectively. Multiple meta-analysis of the relationship between rs12456492 and PD revealed that the G allele and GG and GA genotyping of rs12456492 (A/G) polymorphism may increase PD susceptibility [13, 14]. Recently, the number of the investigations on the association between rs16976358 and ASD is also increased [15–17]. The studies exhibit that the C allele of rs12456492 (T/C) indicates a possible genetic risk for ASD, but not all the genotypes show a significant difference in ASD patients compared to control populations.

For this reason, we carried out a meta-analysis on six genetic models (the allele (C vs. T), codominant (CC vs. TT), codominant (CT vs. TT), dominant (CC+CT vs. TT), recessive (CC vs. CT+TT), and over dominant (TT+CC vs. CT)). We found that CC and CT genotyping of rs16976358 might also underlie the ASD susceptibility in Asian populations. A high heterogeneity was found in all the genetic models in *I*^2^ tests, which could be caused by the heterogeneity of clinical information, methods, and statistics. Moreover, an increasing number of publications could be a possibility to reduce the heterogeneity. To uncover the reason of heterogeneity, the sensitive analyses were further performed in all the 6 models. Results showed the estimated OR in the models of allele (C vs. T), codominant (CC vs. TT), dominant (CC+CT vs. TT), and recessive (CC vs. CT+TT) stayed steadily between the lower and upper CI limits after omitting any of one study, suggesting that the data in most models show stable and reliable results. Collecting numerous of literature may increase the sensitivity in models of codominant (CT vs. TT) and over dominant (TT+CC vs. CT).

Publication bias was not detected in Begg's and Egger's tests but slightly found in the funnel plot when analyzing the genetic models of codominant (CT vs. TT) and dominant (CC+CT vs. TT). This bias could be due to the fact that researchers in Emamalizadeh et al. did not find significant results in the dominant model (CC+CT vs. TT). In addition, it may suggest that the number of studies included in the meta-analysis was not sufficient. Our meta-analysis suggests that the C allele and CC and CT genotyping of rs16976358 polymorphism might be associated with ASD risk, but this results should be verified in the future with a large number of studies and an increasing sample size.

The mechanism of the correlation between *RIT2* rs16976358 and susceptibility to ASD is not well understood. Since SNP rs16976358 is located in the far downstream region of *RIT2* gene, one possibility is that SNP rs16976358 may affect gene and protein expression by altering the distant elements [15]. *RIT2*, as a functional GDP/GTP regulator switch, plays an important role in various signaling pathways within the nervous system. It is involved in EGF receptor- and mSos-mediated signaling pathways and in calcium/calmodulin-mediated cellular processes [18]. Calmodulin regulates numerous Ca^2+^-dependent enzymes and modulates neuronal functions including synaptic development and plasticity, learning, and memory formation [19, 20]. RIT2 signaling is involved in mediating neurogenesis, neuronal differentiation, and neurite growth and branching, showing an important role in normal neuronal development. Furthermore, RIT2 interacts with dopamine active transporters (DATs) in the dopamine (DA) signaling which is mediated by protein kinase C (PKC) activation [21]. In this regard, dysregulated expression of DAergic RIT2 would impact striatal function and DA-dependent behaviors such as movement, motivation, and addictive behaviors [22]. Thus, *RIT2* rs16976358 may disrupt the central dopaminergic system, which is closely related to the ASD pathogenesis [23]. In addition, the investigation of causative linked variants between rs16976358 and ASD susceptibility revealed that the allelic changes may affect the regulatory motif of SOX transcription factor, suggesting another possible mechanism in the emerging etiology of ASD [17]. However, further studies need to be carried out to elucidate this mechanism.

Our meta-analysis has some limitations. Firstly, the publications in English were included in our studies. Secondly, the number of literature and sample size was not large. Thirdly, only Asian populations were analyzed in the current meta-analysis, as the RIT2 rs16976358 polymorphism has not been reported in Caucasian populations. Lastly, despite publication of year and region of patients, we could not analyze the other potential influencing factors due to the insufficiency of data. All these limitations could cause bias in the results, though we try to eliminate the bias.

In conclusion, our meta-analysis indicates that *RIT2* rs16976358 polymorphism is significantly associated with ASD in Asian populations. The allele C, CC, and CT genotyping of rs16976358 suggest the risk of ASD. However, more additional studies from different countries and ethnic populations may be helpful to supply a more reliable estimation of the association between rs16976358 and ASD susceptibility.

## Figures and Tables

**Table 1:** Characteristics of three studies included in this meta-analysis.

Author	Year	Study type	Region	Ethnicity	Genotyping method	Sample size	
						Case	Control
Liu et al.	2016	Case control	Japan and Taiwan	Asian	TaqMan genotyping platform (PCR)	158	425
Hamedani et al.	2017	Case control	Iran	Asian	Amplification refractory mutation system polymerase chain (ARMS-PCR)	532	472
Emamalizadeh et al.	2017	Case control	Iran	Asian	Polymerase chain reaction-restriction fragment length polymorphism (PCR-RFLP)	470	470

**Table 2:** Genotyping distribution and minor allele frequency in this meta-analysis.

Article	Cases					Controls					MAF		Total number of alleles	
	CC	CT	TT	Case	T allele	CC	CT	TT	C allele	T allele	Case	Control	Case	Control
Liu et al.	10	35	112	55	259	9	61	345	79	751	0.212	0.105	316	850
Hamedani et al.	35	231	266	301	763	9	139	324	157	787	0.394	0.199	1064	944
Emamalizadeh et al.	18	117	335	153	787	4	106	360	114	826	0.194	0.138	940	940

**Table 3:** A summary of odds ratio (OR), lower and upper limits of 95% confidence interval (95% CI LL and 95% CI UL), and *p* value in six genetic models ((the allele (C vs. T), codominant (CC vs. TT), codominant (CT vs. TT), dominant (CC+CT vs. TT), recessive (CC vs. CT+TT), and over dominant (TT+CC vs. CT)) from three literatures.

Article	Genetic models	OR	95% CI LL	95% CI UL	*p* value
Liu et al.	Allele C vs. T	2.018719	1.363121	2.972295	0.0003
	Codominant CC vs. TT	3.422619	1.212021	9.752888	0.0125
	Codominant CT vs. TT	1.767418	1.070899	2.882576	0.0217
	Dominant CC+CT vs. TT	1.98023	1.252667	3.107926	0.0023
	Recessive CC vs. CT+TT	3.068783	1.094061	8.700283	0.018
	Over dominant TT+CC vs. CT	0.6006457	0.3696734	0.9876966	0.0335

Hamedani et al.	Allele C vs. T	1.977502	1.583365	2.474171	0.0001
	Codominant CC vs. TT	4.736842	2.178329	11.38494	0.0001
	Codominant CT vs. TT	2.024233	1.53875	2.664732	0.0001
	Dominant CC+CT vs. TT	2.189189	1.677242	2.859186	0.0001
	Recessive CC vs. CT+TT	3.622848	1.682269	8.655594	0.0003
	Over dominant TT+CC vs. CT	0.5439075	0.4148075	0.7125781	0.0001

Emamalizadeh et al.	Allele C vs. T	1.408614	1.075953	1.847109	0.0119
	Codominant CC vs. TT	4.835821	1.568411	19.80614	0.0020
	Codominant CT vs. TT	1.186145	0.8670112	1.623387	0.2817
	Dominant CC+CT vs. TT	1.31886	0.9742033	1.786854	0.0744
	Recessive CC vs. CT+TT	4.639381	1.510498	18.96414	0.0039
	Over dominant TT+CC vs. CT	0.8786043	0.6427384	1.200468	0.4433

**Table 4:** Meta-analysis of rs16976358 polymorphism and risk of ASD in Asian populations. *Q*-test and *I*^2^ are applied to assess the heterogeneity. *Q*-test was used for evaluating *p* value. If *P*_*Q*_ > 0.10, the fixed-effect model was adopted to calculate the pooled ORs; if *P*_*Q*_ < 0.10, the random-effect model was applied instead.

Genetic models	*P*_*Q*_	*I* ^2^	Random model (95% CI)	*P*_*z*_	Fixed model (95% CI)	*P*_*z*_
Allele C vs. T	0.110	85.6%			1.767 (1.517-2.057)	0.001
Codominant CC vs. TT	0.840	72.9%			4.402 (2.630-7.369)	0.001
Codominant CT vs. TT	0.031	92.4%	1.614 (1.127-2.311)	0.009		
Dominant CC+CT vs. TT	0.034	92.1%	1.781 (1.270-2.498)	0.001		
Recessive CC vs. CT+TT	0.849	72.9%			3.700 (2.217-6.176)	0.001
Over dominant TT+CC vs. CT	0.057	90.8%	0.663 (0.479-0.916)	0.013		

**Table 5:** Metaregression of rs16976358 polymorphism and risk of ASD in Asian populations. Publication year and region were analyzed as possible covariates.

Genetic models	*t*	*P* > |*t*|	95% conf. interval
Allele C vs. T	-0.56	0.677	-4.343134-3.97891
Codominant CC vs. TT	0.58	0.664	-6.884959-7.548125
Codominant CT vs. TT	-0.25	0.842	-6.479227-6.226143
Dominant CC+CT vs. TT	-0.32	0.806	-6.122942-5.826483
Recessive CC vs. CT+TT	0.43	0.740	-6.927886-7.416656
Over dominant TT+CC vs. CT	0.30	0.817	-5.656667-5.92586

**Table 6:** Sensitive analysis of rs16976358 polymorphism and risk of ASD in Asian populations. OR, 95% confidence interval, *Z* and *p* value were listed in the six genetic models.

Genetic models	Study	Estimate OR	95% conf. interval	*Z*	*p* value
Allele C vs. T	Liu et al.	1.683	1.207 -2.345	3.071	0.002
	Hamedani et al.	1.644	1.160-2.331	2.791	0.005
	Emamalizadeh et al.	1.988	1.648-2.398	7.181	0.001

Codominant CC vs. TT	Liu et al.	4.768	2.569-8.852	4.949	0.001
	Hamedani et al.	3.954	1.951-8.013	3.814	0.001
	Emamalizadeh et al.	4.164	2.325-7.458	4.797	0.001

Codominant CT vs. TT	Liu et al.	1.557	0.922-2.629	1.658	0.097
	Hamedani et al.	1.389	0.948-2.036	1.685	0.092
	Emamalizadeh et al.	1.958	1.554-2.467	5.704	0.001

Dominant CC+CT vs. TT	Liu et al.	1.707	1.039-2.805	2.112	0.035
	Hamedani et al.	1.565	1.056-2.319	2.232	0.026
	Emamalizadeh et al.	2.132	1.708-2.661	6.698	0.001

Recessive CC vs. CT+TT	Liu et al.	3.918	2.120-7.243	4.357	0.001
	Hamedani et al.	3.644	1.803-7.363	3.603	0.001
	Emamalizadeh et al.	3.393	1.903-6.049	4.141	0.001

Over dominant TT+CC vs. CT	Liu et al.	0.687	0.430-1.099	-1.565	0.118
	Hamedani et al.	0.758	0.527-1.090	-1.494	0.135
	Emamalizadeh et al.	0.557	0.444-0.700	-5.028	0.001

**Table 7:** Publication bias of this meta-analysis using Begg's and Egger's tests.

Genetic models	Test	*z*/*t*	95% conf. interval	*p* value
Allele C vs. T	Begg's test	0.01		1.000
	Egger's test	0.01	-74.14091-74.16632	0.999

Codominant CC vs. TT	Begg's test	0.01		1.000
	Egger's test	-0.17	-27.95273-27.20531	0.891

Codominant CT vs. TT	Begg's test	0.01		1.000
	Egger's test	-0.10	-89.08547-87.70071	0.937

Dominant CC+CT vs. TT	Begg's test	0.01		1.000
	Egger's test	-0.06	-95.84668-94.93263	0.961

Recessive CC vs. CT+TT	Begg's test	0.01		1.000
	Egger's test	0.52	-22.72579-24.66295	0.695

Over dominant TT+CC vs. CT	Begg's test	0.01		1.000
	Egger's test	0.08	-79.20644-80.27258	0.946

## Data Availability

The data used to support the findings of this study are included within the article.
